# Supplementary material for: Assessment of methods used for 3-dimensional superimposition of craniofacial skeletal structures: a systematic review
Source: PeerJ. 2020 Jun 5;8:e9263. doi: 10.7717/peerj.9263 (PMC7278889; doi:10.7717/peerj.9263)
Supplement: Supplemental Information 1 [file peerj-08-9263-s001.docx]

For systematic reviews / meta-analyses, authors are asked to provide the following information:

1. The rationale for conducting the systematic review

Response: The purpose of the present study is to summarize and critically assess the available evidence from studies on skeletal-tissue superimpositions of serial craniofacial CT or CBCT images used to assess any morphological change. Various relevant studies have been published so far, but the heterogeneity of the protocols, machines, acquisition parameters, and superimposition references did not allow for the development of solid conclusions (Ponce-Garcia et al., 2018). Hence, the purpose of this review is to provide a synopsis and a thorough assessment of the current evidence, aiming to provide guidelines for the proper use of the techniques and interpretation of the outcomes and identify areas where further research is needed.

1. The contribution that the systematic review makes to knowledge in light of previously published related reports, including other meta-analyses and systematic reviews.

Response: The only existing systematic evaluation of the literature included studies that were published prior to 2017 and regarded only the anterior cranial base (Ponce-Garcia et al., 2018). Thus, neither the accuracy, the precision, and the reproducibility of hard-tissue superimposition techniques nor the choice of reference structures have been thoroughly investigated recently. The present systematic review included a higher number of studies (15 studies instead of 6), tested a broader topic and provided a thorough, up-to-date, and critical assessment of the available evidence on a highly expanding 3D technique, both in research and in clinical practice. Still following the increased amount of data on the topic, most of the available studies had methodological shortcomings and high applicability concerns. At the moment, certain voxel-based and surface-based superimpositions seem to work properly and to be superior to landmark-based superimposition. However, there is an urgent need for further research in the field to develop and properly validate these techniques on different samples, through high quality studies with low applicability concerns.
