# Supplementary material for: Assessment of methods used for 3-dimensional superimposition of craniofacial skeletal structures: a systematic review
Source: PeerJ. 2020 Jun 5;8:e9263. doi: 10.7717/peerj.9263 (PMC7278889; doi:10.7717/peerj.9263)
Supplement: Supplemental Information 2 [file peerj-08-9263-s002.docx]

**Appendix 1.** Detailed description of the search performed in various databases.

| **Database** | **Search** | **Criteria & Results** |
| --- | --- | --- |
| PubMed  <https://www.ncbi.nlm.nih.gov/pubmed/> | (((((head OR heads OR craniofacial OR cranial OR cranium OR mandib* OR maxilla* OR (cranial base) OR zygoma*)))) AND (superimpos*[Title] OR registrat*[Title)) AND ((((computed tomography) OR (computerized tomography) OR (cone beam tomography) OR (CBCT) OR (three-dimensional CT) OR (three dimensional CT) OR (3-dimensional CT) OR (3D CT) OR (CT scan) OR (CT scans) OR (quantitative CT) OR (qualitative CT) OR (CT analysis) OR (tomography analysis)))) | Publication date:  Up to 2019/11/17  Limits: Humans  Search Builder: All Fields  Results: 509 Matches  Inclusion: 17 Studies |
| EMBASE  <https://www.embase.com/#search> | (head OR heads OR craniofacial OR cranial OR cranium OR mandib* OR maxilla* OR (cranial AND base) OR zygoma*) AND (superimpos* OR registrat*) AND (computed AND tomography OR (computerized AND tomography) OR (cone AND beam AND tomography) OR cbct OR ('three dimensional' AND ct) OR (three AND dimensional AND ct) OR ('3 dimensional' AND ct) OR (3d AND ct) OR (ct AND scan) OR (ct AND scans) OR (quantitative AND ct) OR (qualitative AND ct) OR (ct AND analysis) OR (tomography AND analysis)) AND (2000:py OR 2001:py OR 2002:py OR 2003:py OR 2004:py OR 2005:py OR 2006:py OR 2007:py OR 2008:py OR 2009:py OR 2010:py OR 2011:py OR 2012:py OR 2013:py OR 2014:py OR 2015:py OR 2016:py OR 2017:py OR 2018:py OR 2019:py) AND 'human'/de AND 'article'/it AND ([adolescent]/lim OR [adult]/lim OR [school]/lim OR [young adult]/lim) | Publication date: Up to 2019/11/17  Limits: Humans  Search Builder: All Fields  Special function used: No  Results: 698 Matches  Inclusion: 10 Studies |
| Google Scholar  <https://scholar.google.ch> | With the exact phrase: 3D superimposition  With at least one of the words: head OR heads OR craniofacial OR cranial OR vs OR cranium OR and OR mandible* OR maxilla* OR “cranial base” OR zygoma* | Publication date: Up to 2019  Limits: Humans  Search Builder: All Fields  Results: 797 Matches (First 500 studies screened)  Inclusion: 13 Studies |
|  | With the exact phrase: 3D registration  With at least one of the words: head OR heads OR craniofacial OR cranial OR vs OR cranium OR and OR mandible* OR maxilla* OR “cranial base” OR zygoma* | Publication date:  Up to 2019/11/17  Limits: Humans  Search Builder: All Fields  Results: 15'400 Matches (First 500 studies screened)  Inclusion: 1 study |
| Cochrane Library  <https://www.cochranelibrary.com> | head or heads or craniofacial or cranial or cranium or mandible* or maxilla* or "cranial base" or zygoma or superimpos* or registrat* or "computerized tomography" or "cone beam tomography" or CBCT or "three-dimensional CT" or "three dimensional CT" or "3-dimensional CT" or "3D CT" or "CT scan" or "CT scans" or "quantitative CT" or "qualitative CT" or "CT analysis" or "tomography analysis" | Publication date:  Up to 2019/11/17  Limits: Humans  Search Builder: All Fields  Results: 536 Matches  Inclusion: 0 Studies |
| OpenGrey  <http://opengrey.eu> | 3D superimposition, 3-D superimposition, 3 Dimensional superimposition, 3-Dimensional superimposition, three-dimensional superimposition, 3D registration, 3-D registration, 3 Dimensional registration, 3-Dimensional registration, three-dimensional registration | Publication date:  Up to 2019/11/17  Results: 0 Matches  Inclusion: 0 Studies |
| GreyLiteratureReport  <http://www.greylit.org> | 3D superimposition, 3-D superimposition, 3 Dimensional superimposition, 3-Dimensional superimposition, three-dimensional superimposition, 3D registration, 3-D registration, 3 Dimensional registration, 3-Dimensional registration, three-dimensional registration | Publication date:  Up to 2019/11/17  Search Builder: Full text  Results: 0 Matches  Inclusion: 0 Studies |
