# Supplementary material for: Assessment of methods used for 3-dimensional superimposition of craniofacial skeletal structures: a systematic review
Source: PeerJ. 2020 Jun 5;8:e9263. doi: 10.7717/peerj.9263 (PMC7278889; doi:10.7717/peerj.9263)
Supplement: Supplemental Information 3 [file peerj-08-9263-s003.pdf]

PRISMA Checklist for systematic review

Section/topic	#	Checklist item	Reported on page #
**TITLE**			Page 1
Title	1	Identify the report as a systematic review, meta-analysis, or both.	1
**ABSTRACT**			Page 2
Structured summary	2	Provide a structured summary including, as applicable: background; objectives; data sources; study eligibility criteria, participants, and interventions; study appraisal and synthesis methods; results; limitations; conclusions and implications of key findings; systematic review registration number.	2
**INTRODUCTION**			Page 4-5
Rationale	3	Describe the rationale for the review in the context of what is already known.	5
Objectives	4	Provide an explicit statement of questions being addressed with reference to participants, interventions, comparisons, outcomes, and study design (PICOS).	Page 6
**METHODS**			Page 5-8
Protocol and registration	5	Indicate if a review protocol exists, if and where it can be accessed (e.g., Web address), and, if available, provide registration information including registration number.	5
Eligibility criteria	6	Specify study characteristics (e.g., PICOS, length of follow-up) and report characteristics (e.g., years considered, language, publication status) used as criteria for eligibility, giving rationale.	5
Information sources	7	Describe all information sources (e.g., databases with dates of coverage, contact with study authors to identify additional studies) in the search and date last searched.	5, 6
Search	8	Present full electronic search strategy for at least one database, including any limits used, such that it could be repeated.	Appendix 1
Study selection	9	State the process for selecting studies (i.e., screening, eligibility, included in systematic review, and, if applicable, included in the meta-analysis).	6, 7
Data collection process	10	Describe method of data extraction from reports (e.g., piloted forms, independently, in duplicate) and any processes for obtaining and confirming data from investigators.	7
Data items	11	List and define all variables for which data were sought (e.g., PICOS, funding sources) and any assumptions and simplifications made.	6, 7
Risk of bias in individual studies	12	Describe methods used for assessing risk of bias of individual studies (including specification of whether this was done at the study or outcome level), and how this information is to be used in any data synthesis.	8
Summary measures	13	State the principal summary measures (e.g., risk ratio, difference in means).	6
Synthesis of results	14	Describe the methods of handling data and combining results of studies, if done, including measures of consistency (e.g., I^2) for each meta-analysis.	NA

PRISMA Checklist for systematic review

Section/topic	#	Checklist item	Reported on page #
Risk of bias across studies	15	Specify any assessment of risk of bias that may affect the cumulative evidence (e.g., publication bias, selective reporting within studies).	7
Additional analyses	16	Describe methods of additional analyses (e.g., sensitivity or subgroup analyses, meta-regression), if done, indicating which were pre-specified.	7, 8
**RESULTS**			Page 8 - 14
Study selection	17	Give numbers of studies screened, assessed for eligibility, and included in the review, with reasons for exclusions at each stage, ideally with a flow diagram.	8
+Study characteristics	18	For each study, present characteristics for which data were extracted (e.g., study size, PICOS, follow-up period) and provide the citations.	Tables 2, 3, 4
Risk of bias within studies	19	Present data on risk of bias of each study and, if available, any outcome level assessment (see item 12).	Table 1
Results of individual studies	20	For all outcomes considered (benefits or harms), present, for each study: (a) simple summary data for each intervention group (b) effect estimates and confidence intervals, ideally with a forest plot.	Table 4
Synthesis of results	21	Present results of each meta-analysis done, including confidence intervals and measures of consistency.	NA
Risk of bias across studies	22	Present results of any assessment of risk of bias across studies (see Item 15).	8
Additional analysis	23	Give results of additional analyses, if done (e.g., sensitivity or subgroup analyses, meta-regression [see Item 16]).	NA
**DISCUSSION**			Page 14-17
Summary of evidence	24	Summarize the main findings including the strength of evidence for each main outcome; consider their relevance to key groups (e.g., healthcare providers, users, and policy makers).	15-17
Limitations	25	Discuss limitations at study and outcome level (e.g., risk of bias), and at review-level (e.g., incomplete retrieval of identified research, reporting bias).	Table 5 , 16, 17
Conclusions	26	Provide a general interpretation of the results in the context of other evidence, and implications for future research.	17
**FUNDING**			Page 22
Funding	27	Describe sources of funding for the systematic review and other support (e.g., supply of data); role of funders for the systematic review.	NA

From: Moher D, Liberati A, Tetzlaff J, Altman DG, The PRISMA Group (2009). Preferred Reporting Items for Systematic Reviews and Meta-Analyses: The PRISMA Statement. PLoS Med 6(7): e1000097. doi:10.1371/journal.pmed1000097

For more information, visit: **www.prisma-statement.org**.

PRISMA item # 3

Page 5

Various relevant studies have been published so far, but the heterogeneity of the protocols, machines, acquisition parameters, and superimposition references did not allow for the development of solid conclusions (Ponce-Garcia et al., 2018). The only existing systematic evaluation of the literature included studies that were published prior to 2017 and regarded only the anterior cranial base (Ponce-Garcia et al., 2018). Thus, neither the accuracy, the precision, and the reproducibility of hard-tissue superimposition techniques nor the choice of reference structures have been thoroughly investigated recently.

PRISMA item # 4 and #6

Page 5

• Study design: Any study design, including prospective, and retrospective studies of any type.

• Study sample: Studies with sample size ≥ 3.

• Index test: 3D skeletal-tissue superimposition techniques to assess any change in the morphology of the craniofacial complex.

• Types of participants: Serial craniofacial CT or CBCT images of individuals or skulls who have received any kind of actual or simulated treatment, or whose craniofacial morphology is expected to be altered due to growth or pathology.

• Type of intervention: 3D skeletal-tissue superimposition to assess any morphological change in the craniofacial complex.

• Primary outcome: Superimposition accuracy or precision of a technique, or agreement between techniques measured in terms of angles or distances between specific skeletal or facial landmarks or area distances between corresponding models. Volume differences measured following 3D superimposition were also considered. Studies that evaluated any of the above parameters as a secondary outcome were also included.

• Comparator/control group: Studies that compared different superimposition techniques, direct measurements, or repeated measurements were selected.

• Unit of analysis: The measured distance, angle, or volume.

• Follow-up: Any observation period between subsequent models.

• Exclusion criteria: None.

PRISMA item # 5

The protocol was registered in PROSPERO prior to the study implementation (ID: CRD42019143356).

PRISMA item # 7

The following databases were searched for eligible studies: Medline (via Pubmed), EMBASE, Google Scholar, Cochrane Library, OpenGrey and GreyLiteratureReport. The last search was performed on 17.11.2019, without time restriction. Unpublished literature was searched through the National Research Register, Pro-Quest Dissertation Abstracts and Thesis database, additional hand searches of all relevant studies were also performed. The specific search strategies applied for each database are provided as supplementary information (Appendix 1).

PRISMA item # 8

Appendix 1

PRISMA item # 9

Page 6, 7

Following the search strategy, the selected databases were screened by two authors of the review (Daniel Dinh-Phuc Mai and Sven Stucki). There was no blinding concerning the authors' names and affiliations, or the outcomes of the included studies. Titles and abstracts were evaluated first, if necessary the full text was read to evaluate the eligibility. The same authors read all eligible studies again in full text, independently, whereas non-eligible studies were excluded. Thereafter the eligibility was discussed between all team members until a consensus was reached, under the guidance of the last author (Nikolaos Gkantidis). A record of all decisions made during this process was retained.

PRISMA item # 10 and 11

Page 7

The first and the last author performed data extraction independently and in duplicate, aiming to extract from the eligible studies the following information:

• Methods: Author, title, year, objectives, and design of study.

• Participants: Patient number, age, and gender.

• Materials: 3D model acquisition method and time between serial models.

• Superimposition method: Type of superimposition reference areas or points and software with specific settings used.

• Comparison/control group: Type and characteristics.

• Outcome: Type of outcome(s) and method of outcome assessment.

If necessary, the authors were contacted by email to request missing data. If the relevant information was not provided, only the available information was used.

PRISMA item # 12

Page 8

The quality of the selected studies was evaluated using the QUADAS-2 tool (Whiting, 2011). This is a widely used tool to evaluate the diagnostic accuracy of methods in systematic reviews. Using the QUADAS-2 tool the patient selection, the index test, the reference standard and the flow and timing are evaluated regarding their risk of bias and applicability concerns. Usually, gradings are shown in a table using happy (low risk) or sad smiles (high risk). In case an evaluation is not possible, e.g. because of missing data, an interrogation mark is shown (unclear risk). The total risk of bias or applicability concerns of each study correspond to the worst rating given in the individual items assessed each time.

The quality assessment of all studies was performed by two authors (Daniel Dinh-Phuc Mai and Nikolaos Gkantidis) independently. If there was a disagreement, a consensus was reached through discussion among all authors. Studies graded with a high risk of bias were not to be included in a meta-analysis.

PRISMA item # 13

Page 6

Distance, angle, or volume differences.

PRISMA item # 15

Page 7

We conducted an accurate, but also broad enough search of multiple sources, including on-going studies, to minimize potential reporting biases, such as publication bias and duplicate reports.

PRISMA item # 16

Page 7, 8

Results will also be tested for the following factors, if possible:

• CBCT vs. CT data.

• Growing vs. non-growing patients.

• Short-term (within 1 year) vs. medium/long-term (> 1 year) interval between serial models.

• Superimposition on the anterior cranial base vs. superimposition on maxillary structures vs. superimposition on mandibular structures

PRISMA item # 17

Page 8

The search results are shown in Figure 1. After searching various databases, 2'540 studies were found. Seven additional studies were identified through hand searches. After removing the duplicates, 832 studies remained. These studies were screened by reading the titles and abstracts. Full-text reading of 24 studies was performed to evaluate the eligibility. Nine studies did not match the review question and thus, they were excluded as irrelevant to the study topic. Following the selection process 15 studies were included in this review.

PRISMA item # 22

Page 9

The quality assessment of the included studies is provided in Table 1.

From the 15 included studies, 12 of these have shown a high total risk of bias, one a low risk of bias, and 2 studies have shown an unclear risk of bias. Regarding the individual items 4 studies have high, 7 low, and 4 unclear risk of bias in the patient selection. Regarding the index test, 8 studies have high, 6 low, and one unclear risk of bias. The reference standard of 9 studies shows a high risk of bias, of 2 low, and of 4 unclear. The flow and timing of 2 studies has high, of 11 low, and of 2 unclear risk of bias.

Thirteen out of the 15 studies showed high total applicability concerns, 2 unclear and no study had low applicability concerns. Concerning the individual items, 6 studies had high, 6 unclear and 3 low applicability concerns in the patient selection. Regarding the index test, 9 studies had high, one unclear and 5 low applicability concerns. The reference standard of 9 studies showed high, of 3 unclear and of 3 low applicability concerns.

PRISMA item # 25

Page 17

Though a significant amount of studies was identified, a limitation of the present study is that the heterogeneity of the included studies is high, and the quality of the available evidence is limited. This can be attributed to the fact that the field has been developed in the last few years and gained much attention only recently.

PRISMA item # 26

Page 17

The fast evolution of 3D superimposition techniques has provided a key element in the toolkit of relevant fields to evaluate craniofacial changes following growth or treatment. Due the high heterogeneity and the moderate to low quality of the included studies, few valid conclusions can

be drawn. Most of the available studies had methodological shortcomings and high applicability concerns. Therefore, no clear recommendation could be given at present for proper methods used for 3D-superimposition of craniofacial skeletal structures. At the moment, certain voxel-based and surface-based superimpositions seem to work properly and to be superior compared to landmark-based superimposition. However, further research is necessary to develop and properly validate these techniques on different samples, through studies with high quality and low applicability concerns.
